# Geographically weighted regression analysis for nonnegative continuous outcomes: An application to Taiwan dengue data

**DOI:** 10.1371/journal.pone.0315327

**Published:** 2024-12-12

**Authors:** Vivian Yi-Ju Chen, Yun-Ciao Yang

**Affiliations:** 1 Department of Statistics, National Chengchi University, Taipei, Taiwan; 2 Department of Statistics, Tamkang University, Tamsui, Taipei, Taiwan; Cairo University, EGYPT

## Abstract

Geographically Weighted Regression (GWR) has gained widespread popularity across various disciplines for investigating spatial heterogeneity with respect to data relationships in georeferenced datasets. However, GWR is typically limited to the analysis of continuous dependent variables, which are assumed to follow a symmetric normal distribution. In many fields, nonnegative continuous data are often observed and may contain substantial amounts of zeros followed by a right-skewed distribution of positive values. When dealing with such type of outcomes, GWR may not provide adequate insights into spatially varying regression relationships. This study intends to extend the GWR based on a compound Poisson distribution. Such an extension not only allows for exploration of relationship heterogeneity but also accommodates nonnegative continuous response variables. We provide a detailed specification of the proposed model and discuss related modeling issues. Through simulation experiments, we assess the performance of this novel approach. Finally, we present an empirical case study using a dataset on dengue fever in Tainan, Taiwan, to demonstrate the practical applicability and utility of our proposed methodology.

## Introduction

Spatial heterogeneity, which refers to the instability of data relationships between dependent and independent variables, is recognized as a crucial factor in spatial data analysis [1–3]. This recognition has sparked interest in spatially varying coefficient models. Geographically weighted regression (GWR) has emerged as a popular tool, pioneered by [4]. With a nonparametric modeling framework, GWR employs kernel-based density functions centered on each observation to estimate local parameters, utilizing geographical weights determined by distance. Due to its simplicity and interpretability, numerous extensions of GWR have been developed to address complex data structures and analytical challenges. These extensions include methods for spatiotemporal data (e.g., [5, 6]), count outcomes (e.g., [7, 8]), and approaches dealing with spatial autocorrelation and relationship heterogeneity (e.g., [9, 10]). Significant advancements have also been made in those for quantile versions [3, 11], multivariate responses [12], machine learning techniques (e.g., [13, 14]), and models capable of handling zero-inflated data [15].

This paper aims to enhance the development of Geographically Weighted Regression (GWR) by introducing a flexible approach for modeling nonnegative continuous data that may exhibit severe skewness and a substantial proportion of zeros. Such data, also called semi-continuous variables, arise frequently in various fields, including health services (e.g., medical costs), environmental sciences (e.g., daily precipitation levels), ecology (e.g., total weight of a particular fish species), social studies (e.g., alcohol consumption), and actuarial research (e.g., insurance claim losses), among others. In spatial applications, it is not unusual for researchers to be interested in analyzing semi-continuous outcomes. For example, Reich et al. [16] investigated the spatial impact of neighborhood environmental characteristics on the physical activity levels of pregnant women across different regions. Their study focused on activity intensity, a semi-continuous variable characterized by a large number of zero values (indicating no activity during pregnancy) and highly right-skewed positive, continuous values (indicating varying levels of activity among exercising pregnant women). Modeling such spatial semi-continuous outcomes is challenging due to the point mass at zero and the presence of positive heavy-tailed continuous values. Traditional spatial models based on normal, gamma, and log-normal distributions can yield biased estimates and incorrect inferences when applied to such data [17–19].

Given the mixture of zero and non-zero values, semi-continuous data are often analyzed using two-part models, which involve a two-stage process: one governing the occurrence of zeros and another determining the observed value given a positive non-zero continuous response [17–19]. Several developments have extended two-part models for application to spatial semi-continuous data. Dreassi et al. [20] developed a hierarchical Bayesian approach that constructs a two-part random effect model for small area estimations and handles semi-continuous, skewed, spatially structured data. Neelon et al. [21] proposed a broad class of Bayesian two-part models in which various parametric and nonparametric modeling specifications can be considered for the spatial density estimation of semi-continuous data. More recently, Paradinas et al. [22] focused on spatiotemporal extensions for two-part models to infer the spatiotemporal behavior of the process under study. While quite appealing, these spatial two-part modeling approaches share some disadvantages in practical data analysis.

On one hand, spatial two-part modeling approaches explicitly separate zeros and positive values using two submodels: a binary model (typically logistic regression) that estimates the probability of the outcome being positive in Part I, and a (generalized) linear regression model that estimates the amount of the (transformed) positive value in Part II [21, 23]. One important issue in such model specification is determining the distributional form in Part II. Various choices are suggested in the literature for modeling the positive component, including the lognormal model, log-skewed normal model, and generalized gamma model. An orthodox approach can fit all of these models for a particular dataset and then select the best one based on measures of goodness of fit. However, this approach requires specific fitting algorithms for each candidate model, which can pose computational challenges in empirical applications. Additionally, analysts may struggle to confidently select the most suitable model because different criteria for model comparison can lead to different selected models. On the other hand, spatial two-part models allow for two sets of covariates in the two parts of the model. However, such a feature raises concerns about how to define the predictors to be considered. The number of predictor variables also plays a role. Although one could assume identical predictors for both parts of the model, adding an additional linear function in Part I increases model complexity and makes interpretation of the results more difficult. Another option is to use different independent variables in the two equations, but doing so requires conceptual reasons or appropriate justifications.

Drawing from previous discussions, we advocate a unified approach that enables simultaneous modeling of both zero and positive observations without splitting them into separate parts. In statistics, the compound Poisson model (termed CPM for simplicity) presents an interesting alternative for the analysis of semi-continuous data. This modeling technique assumes that the response variable follows a single distribution known as compound Poisson distribution, which belongs to the Tweedie exponential dispersion family [24, 25]. The CPM has a unique parameter characterizing the power relation between distribution mean and variance. As such, it offers flexibility in modeling continuous response data with large variances and/or excessive zeros [26]. Moreover, it has shown that CPM is comparable in model fit with the two-part models but only includes a one-stage modeling process, thereby enhancing interpretability [27]. Owing to these advantages, several scientists have adopted the modeling for spatial data analysis. For instance, Arcuti et al. [28] introduced a spatiotemporal generalized additive model that draws upon the compound Poisson distributional assumption to deal with spatial zero-inflated abundance data. Swallow et al. [19] developed a Bayesian hierarchical model utilizing the compound Poisson distribution to directly handle the excess number of zeros in nonnegative continuous data while also addressing spatial and temporal correlation. Despite these advancements, little effort has been made to extend the CPM with a local spatial modeling perspective for properly exploring spatially varying relationships with semi-continuous responses, particularly on the GWR framework.

Our motivating example herein is the analysis of dengue data in Taiwan during the 2015 calendar year. The variable of interest is the dengue intensity index, which measures the severity of dengue transmission. This index is a nonnegative continuous variable that can exhibit a point mass at zero and a heavily right-skewed distribution of positive values. Since dengue data are typically georeferenced, spatial models that account for these data characteristics are of interest when estimating the dengue intensity indices in relation to regional covariates such as socioeconomic, demographic, or environmental factors. As it is essential to consider local impacts in dengue epidemiology [29], traditional GWR assumes a normal distribution for the outcome variable at each local calibration and can not effectively address the issues of zero-inflation and heavy skewness in the data. Consequently, it fails to provide appropriate information about spatial heterogeneity. We intend to tackle the analytical challenges by proposing a more suitable approach for dengue data analysis.

This study fills the methodology gap and demonstrates an extension of CPM to GWR by proposing a novel approach named geographically weighted compound Poisson regression model (GWCPM). We begin with a brief review of non-spatial CPM and then present the framework of GWCPM, covering aspects such as bandwidth selection, statistical inferences, and tests of spatial nonstationarity. Simulation experiments are conducted to validate the proposed methodology. Finally, we apply GWCPM to the empirical data on dengue fever in Taiwan, demonstrating its practical utility. The paper concludes with a discussion of the findings and implications.

## Methodology

### A non-spatial CPM

Let *Y*_*i*_ be a response for *i*th observation, *i* = 1, …, *n*, and **X**_*i*_ = [*X*_*i*1_, …, *X*_*ic*_] denote a row vector of covariates with dimension (1 × *c*) for *i*th observation, which includes the intercept. According to Jøregenson [24], the non-spatial CPM is a statistical model where the response variable is assumed to follow the compound Poisson (CP) distribution, denoted as *Y*_*i*_ ∼ CP(*μ*_*i*_, *ϕ*, *p*). This three-parameter distribution can be expressed as

$$\begin{array}{c} f_{Y}\left(y_{i};\mu_{i},\phi ,p\right)=a\left(y_{i};\phi ,p\right)\text{exp}(\frac{1}{\phi }(\frac{y_{i}\mu_{i}^{1-p}}{1-p}-\frac{\mu_{i}^{2-p}}{2-p})), \end{array}$$ (1)

for *y*_*i*_ ≥ 0 where

$$\begin{array}{c} a\left(y_{i};\phi ,p\right)=\{\begin{array}{cc} \sum_{r=1}^{\infty }\frac{1}{r!\Gamma (r\alpha )y}(\frac{{\left(1/\phi \right)}^{\alpha +1}y^{\alpha }}{{\left(p-1\right)}^{\alpha }\left(2-p\right)}{)}^{r}, & y_{i}>0\\ 1, & y_{i}=0 \end{array} \end{array}$$ (2)

with Γ being the gamma function and *α* = (2 − *p*)/(1 − *p*). The distribution mean *μ*_*i*_ is typically modeled based on the covariate vector **X**_*i*_ with the associated regression parameters ***β*** = [*β*_1_, …, *β*_*c*_]^*t*^ (dimension *c* × 1):

$$\begin{array}{c} \mu_{i}=E\left(Y_{i}|{\text{X}}_{i}\right)=g^{-1}\left(\eta_{i}\right)=g^{-1}\left({\text{X}}_{i}\beta \right) \end{array}$$ (3)

for a known monotonic link function *g* that relates *μ*_*i*_ to the linear predictor *η*_*i*_ = *g*(*μ*_*i*_) = **X**_*i*_***β***. The variance of *Y*_*i*_ has the form

$$\begin{array}{c} Var\left(Y_{i}\right)=\phi \mu_{i}^{p} \end{array}$$ (4)

which characterizes a so-called power mean-variance relationship [24, 30]. Here, *ϕ* > 0 is the dispersion parameter, and *p* ∈ (1, 2) is a power index parameter that controls the shape of the response distribution. In Eq (2), *a*(*y*_*i*_; *ϕ*, *p*) represents a normalizing function and needs to be evaluated using an infinite series expansion. Further technical information can be found in [30, 31].

**Fig 1:**
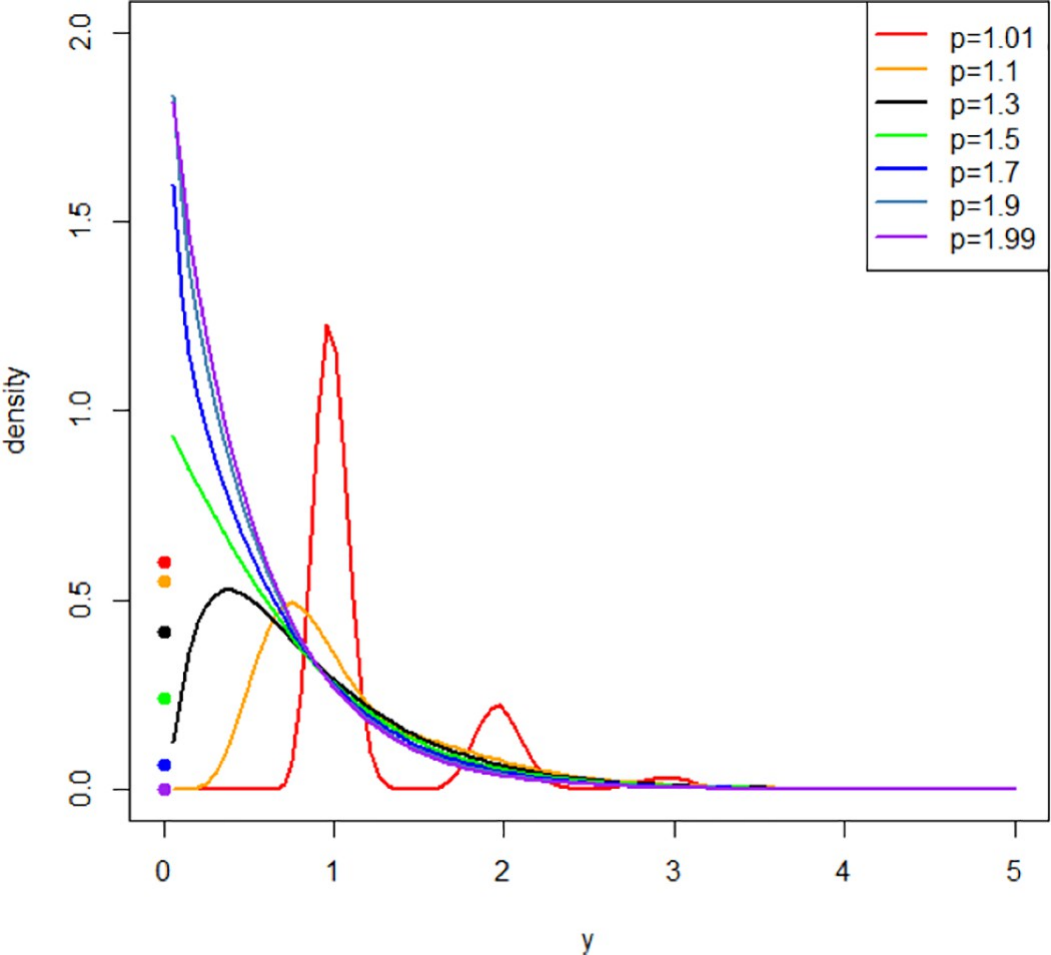
Compound Poisson distribution densities with *μ* = 0.5 and *ϕ* = 1 for different values of *p*.

The variance function of (4) plays an important role in the CP distribution. Depending on the power index parameter *p*, the CP density is a mixed distribution characterized by a positive probability mass at zero and continuous positive support. This feature offers flexibility in modeling various characteristics of nonnegative continuous data. As displayed in Fig 1, the CP densities with mean (*μ*) and dispersion (*ϕ*) parameters fixed at 0.5 and 1, respectively, have different shapes, skewness, variabilities, and discrete probability of *Y* = 0 (solid circles) for various *p* values. In other words, the key advantage of the CP distribution lies in its ability to simultaneously capture both a mass at zero and a unimodal or multimodal distribution with a long right tail. This capability is attributed to the unique specification of the power index parameter *p*, which defines the variance function and characterizes the distribution on varying shapes [24].

### GWCPM specification

We develop GWCPM as an extension of the described non-spatial CPM to GWR. Suppose that we now have data collected from *n* locations in space. Adopting the notations used in previous subsection, let *Y*_*i*_ be a semi-continuous random variable collected from *i*th location (*u*_*i*_, *v*_*i*_), *i* = 1, 2, …, *n*; **X**_*i*_ denote the row vector of *c* georeferenced covariates at location *i*. The GWCPM is then specified by

$$\begin{array}{c} Y_{i}∼\text{CP}[g^{-1}({\text{X}}_{i}\beta \left(u_{i},v_{i}\right)),\phi \left(u_{i},v_{i}\right),p\left(u_{i},v_{i}\right)] \end{array}$$ (5)

where ***β***(*u*_*i*_, *v*_*i*_) indicates the regression coefficient vector at location (*u*_*i*_, *v*_*i*_), such that the mean and variance of *Y*_*i*_ can be expressed as

$$\begin{array}{c} \begin{array}{cc} & E\left(Y_{i}|{\text{X}}_{i},u_{i},v_{i}\right)=\mu_{i}=g^{-1}\left(\eta_{i}\right)=g^{-1}({\text{X}}_{i}\beta \left(u_{i},v_{i}\right))\\ & Var\left(Y_{i}\right)=\phi \left(u_{i},v_{i}\right)\mu_{i}^{p(u_{i},v_{i})} \end{array} \end{array}$$ (6)

for the parameters *ϕ*(*u*_*i*_, *v*_*i*_) and *p*(*u*_*i*_, *v*_*i*_) defined similarly as non-spatial CPM. Expanding upon non-spatial CPM, GWCPM remains the power relationship between the mean and variance. In contrast to the non-spatial CPM, the GWCPM considers spatially varying parameters through *ϕ*(*u*_*i*_, *v*_*i*_), *p*(*u*_*i*_, *v*_*i*_) and ***β***(*u*_*i*_, *v*_*i*_). The vector ***β***(*u*_*i*_, *v*_*i*_) = [*β*_1_(*u*_*i*_, *v*_*i*_), …, *β*_*c*_(*u*_*i*_, *v*_*i*_)]^*t*^ consists of *local* regression coefficients for the explanatory variables **X**_*i*_ evaluated at each location (*u*_*i*_, *v*_*i*_). Thus, the GWCPM can be viewed as a spatial local variant of the non-spatial CPM. It enables users not only to examine the spatial heterogeneity in the data relationships across geographical locations but also to handle spatial semi-continuous outcomes with excessive zeros and severe skewness through the dispersion and index parameters.

It is worth noting that the proposed GWCPM (Eqs 5 and 6) specifies the local index parameter within the range of 1 < *p*(*u*_*i*_, *v*_*i*_) < 2. To enhance its analytical capabilities, we also explore a variant of GWCPM where *p*(*u*_*i*_, *v*_*i*_) can take on arbitrary values beyond this range, but not between 0 and 1. Such relaxation with the unbounded power parameter can automatically adapt to a wider range of variance structures [32], making it more useful than the original GWCPM in handling diverse characteristics of nonnegative continuous data at each local calibration.

### Estimation and inference for GWCPM

By the concepts of GWR, the GWCPM is calibrated by a kernel regression methodology in which the model estimators are obtained in a pointwise way. The model parameters of GWCPM can be estimated using the maximum likelihood estimation method. Let **λ**(*u*_*i*_, *v*_*i*_) = {***β***(*u*_*i*_, *v*_*i*_), *ϕ*(*u*_*i*_, *v*_*i*_), *p*(*u*_*i*_, *v*_*i*_)}. The local log-likelihood function at location *i* is expressed by

$$\begin{array}{ccc} & LL(\lambda (u_{i},v_{i}))=\sum_{j=1}^{n}\{\text{log}\;f_{Y}(y_{j};\mu_{j},\phi \left(u_{i},v_{i}\right),p\left(u_{i},v_{i}\right))\}w_{ij} & \\ & \;=\sum_{j=1}^{n}\{\text{log}[a(y_{j};\phi \left(u_{i},v_{i}\right),p\left(u_{i},v_{i}\right))]+\frac{1}{\phi (u_{i},v_{i})}(y_{j}\psi_{j}-b\left(\psi_{j}\right))\}w_{ij} \end{array}$$ (7)

where $\psi_{j}=\frac{\mu_{j}^{1-p(u_{i},v_{i})}}{1-p(u_{i},v_{i})}$, $b\left(\psi_{j}\right)=\frac{\mu_{j}^{2-p(u_{i},v_{i})}}{2-p(u_{i},v_{i})}$ for *μ*_*j*_ = *g*^−1^(**X**_*j*_***β***(*u*_*i*_, *v*_*i*_)) as the mean evaluating at location *j* with parameters at regression point *i*, and *f*_*Y*_(*y*_*j*_; *μ*_*j*_, *ϕ*(*u*_*i*_, *v*_*i*_), *p*(*u*_*i*_, *v*_*i*_)) represents the CP density distribution of Eq (1) with the parameterizations of Eq (6). The term *w*_*ij*_ = *K*(*d*_*ij*_, *h*) is the geographical weight assigned locally to observation (**X**_*j*_, **Y**_*j*_), calculated based on a kernel function *K* that places more weights on observations closer to (*u*_*i*_,*v*_*i*_) than those farther away. It depends on the distance *d*_*ij*_ between the given location (*u*_*i*_,*v*_*i*_) and the *j*th designed location (*u*_*j*_,*v*_*j*_), as well as a bandwidth *h*. Multiple choices are available for kernel weight schemes in local calibrations. Two prevalent options include fixed gaussian and adaptive bisquare kernels. The fixed gaussian kernel employs a constant bandwidth, ensuring uniform weights between observation points regardless of the distance. In contrast, the adaptive bisquare kernel dynamically adjusts its bandwidth by considering the density of neighboring points surrounding each estimation point with a nearest-neighbor approach. For further details, please refer to Fotheringham et al. [1].

Maximizing the local log-likelihood in (7) at each location involves solving the derivative equations $(\frac{\partial LL(\lambda (u_{i},v_{i}))}{\partial \beta_{1}\left(u_{i},v_{i}\right)},\cdots ,\frac{\partial LL(\lambda (u_{i},v_{i}))}{\partial \beta_{c}\left(u_{i},v_{i}\right)})=0$, $\frac{\partial LL(\lambda (u_{i},v_{i}))}{\partial \phi (u_{i},v_{i})}=0$, and $\frac{\partial LL(\lambda (u_{i},v_{i}))}{\partial p(u_{i},v_{i})}=0$. Since the CP distribution does not have a closed form [30, 31], an iterative maximization algorithm is required to solve the optimization problem numerically. In this paper, we utilize the Quasi-Newton algorithm, which avoids the need for explicitly computing the second derivative (i.e., the Hessian matrix). Instead, it iteratively approximates the Hessian matrix using information from the first derivatives, providing an efficient approach for optimization. By employing the Quasi-Newton approach for parameter estimation, the (numerically) Hessian matrix, denoted as $H(\lambda \left(u_{i},v_{i}\right))$, can be computed at the optimum point. Consequently, the asymptotic distribution of $\hat{\lambda }\left(u_{i},v_{i}\right)$ follows a normal distribution with mean **λ**(*u*_*i*_, *v*_*i*_) and variance $-H{\left(\lambda \left(u_{i},v_{i}\right)\right)}^{-1}$. For testing hypotheses such as *H*_0_: *β*_*k*_(*u*_*i*_, *v*_*i*_) = 0 for *k* = 1, …, *c*, *H*_0_: *ϕ*(*u*_*i*_, *v*_*i*_) = 0, or *H*_0_: *p*(*u*_*i*_, *v*_*i*_) = 0, corresponding local pseudo *t*-statistics can be computed. These statistics serve as tools to evaluate the statistical significance of the local parameters of the GWCPM.

### Bandwidth selection

As mentioned above, local calibrations for GWCPM entail a bandwidth *h* that regulates both the model complexity and the parameter estimates. To determine the optimal bandwidth, we adopt the leave-one-out cross-validation technique suggested by Fotheringham et al. [1]. We attempt to minimize the prediction error, which is calculated as follows:

$$\begin{array}{c} CV\left(h\right)=\sum_{i=1}^{n}(Y_{i}-{\hat{Y}}_{\neq i}\left(h\right){)}^{2} \end{array}$$

where ${\hat{Y}}_{\neq i}\left(h\right)$ is the fitted value of *Y*_*i*_ with calibration location *i* left out of the estimation dataset using bandwidth *h*, and *CV* is termed the cross-validation score. The cross-validation minimization given above is an iterative process wherein the GWCPM is refitted multiple times using the data excluding location *i* (*i* = 1, 2, …, *n*) over a range of bandwidth values. Therefore, numerical search routines for function minimization can be applied to improve efficiency.

### Test of spatial nonstationarity and semiparametric model

For GWCPM, it is essential to identify whether the model parameters (regression coefficients, dispersion parameter, power index parameter) really vary across space. We carry out such assessment using a Monte Carlo randomization testing approach, which has been widely used in GWR literature because it does not require the determination of sampling distribution for the variance of the local parameter estimates. The testing procedure involves the following steps: (i) Calculate the variance of the estimated local parameter estimates for GWCPM using the observed spatial data; (ii) Randomly permute the geographic coordinates of the observations and perform GWCPM for this new configuration; (iii) Calculate the variance of local parameters according to the new estimates; (iv) Repeat the previous two steps for a total of *R* times, say 999; (v) Compute the number of times, say *r*, that the simulated variances obtained from Step (iii) exceed the original value in Step (1). The Monte Carlo *p*-value can be then approximated by *r*/(*R* + 1).

If the Monte Carlo tests yield non-significant results, it suggests that certain parameters of the GWCPM are spatially stationary, while others vary geographically [1, 33]. In response to such scenarios, we introduce a semiparametric model for the analysis of nonnegative continuous data. For cases where only some regression coefficients of independent variables remain constant, we suggest the following semiparametric model:

$$\begin{array}{c} Y_{i}∼\text{CP}[g^{-1}({\text{X}}_{is}\beta_{s}\left(u_{i},v_{i}\right)+{\text{X}}_{iz}\beta_{z}),\phi \left(u_{i},v_{i}\right),p\left(u_{i},v_{i}\right)]. \end{array}$$ (8)

where ***X***_*iz*_ represents a vector of *c*_*z*_ independent variables with constant regression coefficients ***β***_*z*_ and ***X***_*is*_ is defined similarly for a vector of *c*_*s*_ spatially varying parameters; *c* = *c*_*z*_ + *c*_*s*_. A similar model specification can be constructed by further considering global dispersion and/or power parameters. For example, the semiparametric GWCPM with a constant power parameter can be specified as

$$\begin{array}{c} Y_{i}∼\text{CP}[g^{-1}({\text{X}}_{is}\beta_{s}\left(u_{i},v_{i}\right)+{\text{X}}_{iz}\beta_{z}),\phi \left(u_{i},v_{i}\right),p]. \end{array}$$ (9)

Regarding the semiparametric model estimations, we implement a two-stage calibration procedure inspired by the approach of Li and Mei [34]. Using the model in Eq (8) as an example, we first perform a standard GWCPM in which fixed parameters ***β***_*z*_ are treated to vary spatially. We then obtain the final estimators ${\hat{\beta }}_{z}$ by averaging the corresponding local estimates across all locations. In the second stage, another GWCPM is considered to compute refined estimators for the spatially varying coefficients ***β***_*s*_(*u*_*i*_, *v*_*i*_), while keeping the coefficients ${\hat{\beta }}_{z}$ unchanged in the log-likelihood function. This two-stage method can be more effective than the iterative back-fitting approach typically used for mixed GWR models [12, 34].

## Simulation study

We conduct two simulation experiments to assess the estimation accuracy and model performance for both GWCPM and semiparametric GWCPM. The first experiment generates data for GWCPM using the equation

$$Y_{i}∼CP(\mu_{i},p\left(u_{i},v_{i}\right),\phi \left(u_{i},v_{i}\right))$$

where *μ*_*i*_ = exp(*β*_0_(*u*_*i*_, *v*_*i*_) + *β*_1_(*u*_*i*_, *v*_*i*_)*X*_*i*_); *X*_*i*_ are obtained from random draws of the uniform distribution on the interval [0, 1], denoted *X*_*i*_ ∼ *U*(0, 1). Following Chen et al. [3], three sets of real coordinates are used for the geographical system (*u*_*i*_, *v*_*i*_) in the experiment: 159 counties in Georgia, 577 minor civil divisions in Georgia, and 1054 minor civil divisions in North Carolina. The true varying coefficients for each configuration are generated based on the eigenvectors of the transformed binary spatial weight matrix derived from the coordinate system, following the approach proposed by Páez et al. [35]. We select four of them, namely the first (**e**_1_), fourth (**e**_4_), eighth (**e**_4_), and ninth (**e**_9_) eigenvectors, and set

$$\begin{array}{ccc} {\text{e}}_{4} & = & \beta_{0}\left(u_{i},v_{i}\right),\;{\text{e}}_{1}=\beta_{1}\left(u_{i},v_{i}\right)\\ {\text{e}}_{8}+1.5 & = & p\left(u_{i},v_{i}\right),\;{\text{e}}_{9}+1.5=\phi \left(u_{i},v_{i}\right). \end{array}$$

For the semiparametric GWCPM, we extend the data-generating process to include an additional independent variable *X*_2_ with a global constant coefficient *β*_2_. Also, we assume fixed dispersion and power parameters across locations. Specifically, the data is simulated as follows:

$$Y_{i}∼CP(\mu_{i},p,\phi ),\;\mu_{i}=\text{exp}(\beta_{0}\left(u_{i},v_{i}\right)+\beta_{1}\left(u_{i},v_{i}\right)X_{i1}+\beta_{2}X_{i2}).$$

Here, *β*_0_(*u*_*i*_, *v*_*i*_), *β*_1_(*u*_*i*_, *v*_*i*_), and *X*_*i*1_ are specified in the same manner as the first simulation, with *β*_2_ = 1.5, *X*_*i*2_ ∼ *U*(0, 1), *p* = 1.5, and *ϕ* = 0.5.

Throughout the experiments, we employ the fixed gaussian kernel for the model calibrations. A total of 100 simulations are performed for each sample size (*n* = 159, 577, 1054), and the optimal bandwidth is computed using the CV method for each replication. The simulation results are examined with respect to the absolute bias (ABS) and root mean square error (RMSE).

Table 1 presents the results of the mean ABS and RMSE for these two simulation cases. As can be seen, both the mean ABS and RMSE of the *β* regression coefficients for GWCPM decrease with the increase in the sample size, which follows the theoretical expectation. There seems to be a trade-off between the accuracy of estimating the dispersion and power index parameters, where decreasing one tends to increase the other as the sample size grows. This may be consistent with the known fact that the power index parameter can significantly influence the estimation of the dispersion parameter, and they exhibit a reciprocal relationship [26, 36]. Nevertheless, the results are reasonable, and overall, the GWCPM performs well in estimating the true parameters. Similar results are also found for the case of semiparametric GWCPM. This suggests that our proposed two-stage estimation approach has favorable finite sample properties.

**Table 1:** Mean ABS and RMSE over all sampling locations.

Estimates	*n* = 159		*n* = 577		*n* = 1054	
	ABS	RMSE	ABS	RMSE	ABS	RMSE
Case 1: GWCPM						
*β*_0_(*u*, *v*)	0.0617	0.2355	0.0312	0.1264	0.0212	0.1012
*β*_1_(*u*, *v*)	0.0434	0.2109	0.0208	0.1079	0.0158	0.0869
*p*(*u*, *v*)	0.0430	0.0721	0.0529	0.0823	0.0810	0.1481
*ϕ*(*u*, *v*)	0.5658	0.6351	0.5631	0.6285	0.4919	0.5582
Case 2: Semiparametric GWCPM						
*β*_0_(*u*, *v*)	0.0564	0.1491	0.0236	0.0826	0.0182	0.0579
*β*_1_(*u*, *v*)	0.0382	0.1026	0.0196	0.0565	0.0176	0.0411
*β*_2_	0.0023	0.0727	0.0004	0.0392	0.0011	0.0288
*p*	0.2346	0.3028	0.1293	0.2241	0.1821	0.2683
*ϕ*	0.2859	0.3281	0.2191	0.2695	0.2525	0.3003

## Empirical application

### Data and variables

The empirical application involves the analysis of dengue data from Taiwan, an island that has regularly experienced dengue epidemics in recent decades. In 2015, southern Taiwan, particularly Tainan City, experienced the deadliest dengue fever outbreak to date, with greater severity than in other regions. Such geographical disparities highlight the spatial variation in dengue epidemics across different regions, emphasizing the impact of locality. Investigating the spatial nonstationarity of potential risk factors for dengue epidemics is crucial for formulating effective, location-specific dengue control strategies.

To this end, we collect and analyze a dataset of 752 ‘Li’ units in Tainan City for the year 2015. The primary outcome is the dengue index of transmission intensity, a metric developed to assess the severity of epidemic transmission based on the frequency of cases occurring in consecutive weeks. We first obtain dengue case data at the ‘Li’ level from the database maintained by Taiwan’s Centers for Disease Control (Taiwan CDC). We then convert the aggregated dengue counts into an intensity index following the definition of Wen et al. [37, 38]. According to their research, this intensity index is a nonnegative continuous measure that can take on both zero and positive values, rather than being count-based. High values of the index indicate a time-concentrated dengue transmission in the area, while ‘Li’ units with no dengue cases result in zero values for the index.

Building upon previous studies analyzing Taiwan dengue data [39], we consider eight explanatory variables consisting of socio-demographic characteristics, environmental attributes, and meteorological conditions. The socio-demographic factors include population density (*X*_1_), percent of the susceptible population (*X*_2_), and median income (*X*_3_), which are collected from the social-economic platform of the National Geographic Information System maintained by the Department of Statistics, Ministry of Interior. Because both the population density and median income vary quite greatly and are highly skewed, we normalize them with natural logarithm. The Breteau index (BI), extracted through the active vector surveillance system of Taiwan CDC, quantifies the density of immature *Aedes* mosquitoes by measuring the number of positive containers per 100 houses. We calculate the maximum BI at the Li level and use it as an environmental indicator in the analysis. Our last four independent variables are meteorological measures obtained from the Central Weather Bureau in Taiwan: the mean days of most suitable temperature (MST; *X*_5_) for dengue vector mosquitoes, the total number of rainy days from June to November (*X*_6_), the longest duration of dryness days from January to May (*X*_7_), and the annual maximum temperature (*X*_8_). These climatic summaries are spatially extrapolated using the inverse distance weighted (IDW) method to obtain representative results for each ‘Li’ unit. Full descriptive statistics for all variables are given in Table 2.

**Table 2:** Summary statistics of the data.

Variable	Mean	S.D.	Min	Q1	Median	Q3	Max
Outcome (*Y*)	5.70	13.41	0.00	0.00	0.97	4.03	121.08
Logarithm of population density (*X*_1_)	7.34	1.86	2.50	5.90	7.14	9.19	10.79
Percent of susceptible population (*X*_2_)	0.27	0.04	0.17	0.25	0.27	0.30	0.45
Logarithm of median income (*X*_3_)	6.33	0.13	5.93	6.25	6.32	6.39	6.99
Maximum BI (*X*_4_)	1.29	1.35	0.00	0.00	1.00	2.00	5.00
Mean days of MST (*X*_5_)	16.87	4.35	7.27	13.32	16.05	19.76	28.13
Total rainy days from Jun to Nov (*X*_6_)	47.59	3.63	39.44	45.56	47.24	48.64	72.98
Longest duration of dryness days (*X*_7_)	43.10	5.36	32.08	39.23	43.24	46.49	76.42
Annual maximum temperature (*X*_8_)	34.52	0.68	32.26	34.14	34.47	34.79	37.77

**Fig 2:**
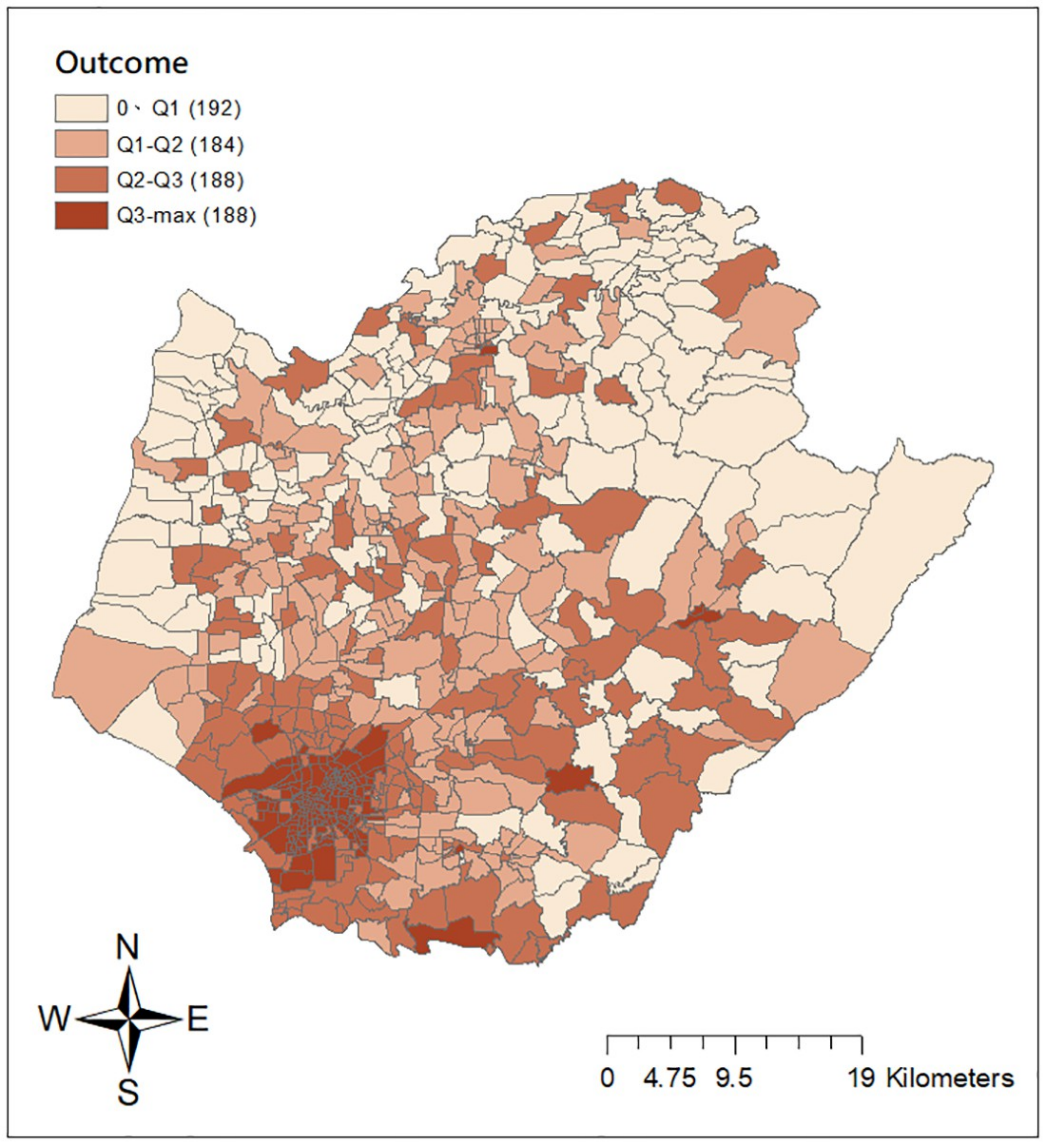
The spatial distribution of dengue transmission intensity at 752 ‘Li’ units in Tainan city for year 2015. Source: The figure is created by the authors in ArcGIS 10.3. The shapefile used to create the map is publicly available from the Taiwan Ministry of the Interior (https://www.tgos.tw); no copyrighted material was used.

**Fig 3:**
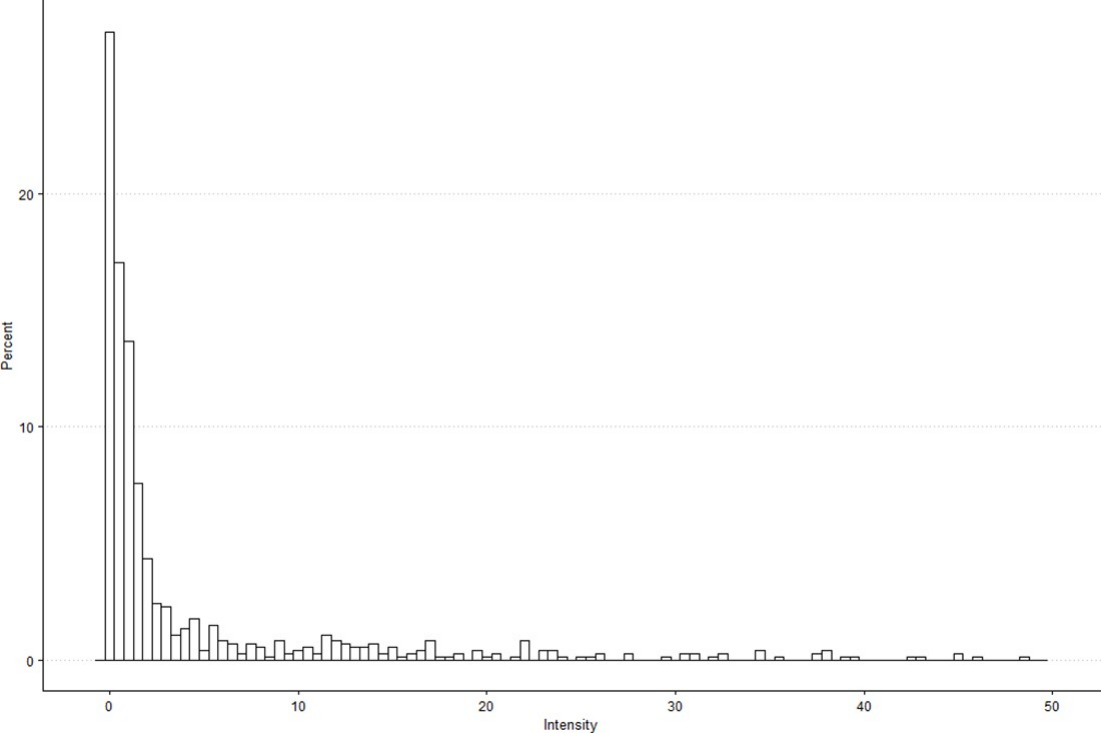
The partial histogram of dengue transmission intensity (up to 60) in Tainan city for year 2015.

The spatial distribution of dengue transmission intensity is given in Fig 2. It finds that ‘Li’ units around the inner downtown area of Tainan (e.g. West Central, North, South, Yongkang, Anping, East districts) with denser populations tend to have higher transmission. Fig 3 depicts the partial histogram for the observed values of the dengue transmission intensity up to 60. There are 10 additional observations above 60 with a maximum value of 132.8. We observe that approximately 26% of the 752 ‘Li’ units in Tainan city reported zero intensity values. The distribution of dengue transmission intensity is highly right-skewed with a cluster at zero and a long tail to the right. The continuous positive intensity values range from 0.13 to 121.08, averaging around 7.66 with a large variance of 226.68. These data characteristics suggest that dengue transmission intensities are potentially zero-inflated with a skewed heavy-tailed distribution of the positive outcomes, which poses challenges for statistical analysis. In particular, conventional data transformations may prove ineffective in normalizing the distribution due to the substantial proportion of zero values, rendering models based on normal distributions inappropriate. Other common models, such as those considered lognormal, gamma, or inverse gaussian distributions, may also be unsuitable as they do not account for zero observations in their modeling process. Consequently, the compound Poisson model emerges as a potential candidate for fitting the dengue transmission intensity data. While recent studies have discussed the relevance of the compound Poisson distribution for infectious disease modeling [40, 41], we highlight the interest in exploring its potential within the framework of GWR for dengue analysis. We use the proposed GWCPM approach to effectively handle the distinctive features of dengue transmission intensity outcomes, namely a considerable proportion of zeros, severe right skewness, and large variance. This approach also allows us not only to identify potential factors of the dengue intensity index but also to examine whether these impacts are stable over space.

### Model results

**Fig 4:**
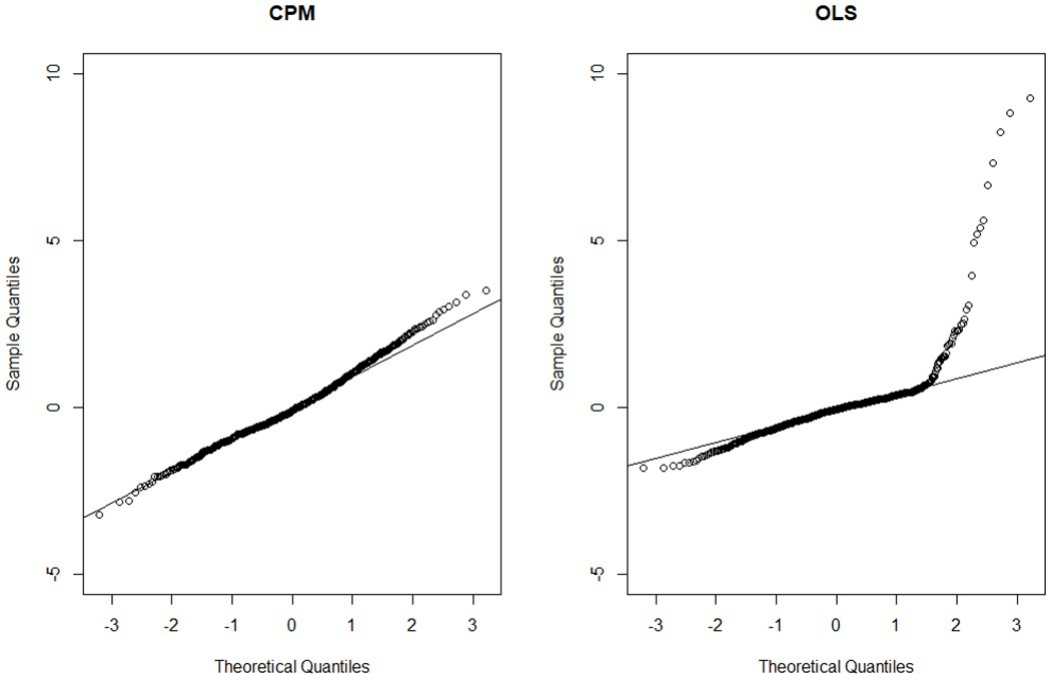
Sample quantile-quantile (QQ) plot of quantile residuals for non-spatial CPM (left panel) and OLS (right panel) models.

We first analyze the data from a global perspective by fitting a non-spatial global CPM as the base model and using an OLS model for comparison. Table 3 shows that the parameter estimates obtained from the two models are quite different, which is in line with our expectations given the different distributional assumptions (CP and normal distributions) of the two models. In both models, the logarithm of median income is negatively associated with dengue intensity; the higher the median income in the area, the lower the intensity of dengue transmission. Conversely, all other variables (excluding intercept) demonstrate positive and statistically significant effects on dengue intensity for CPM. The OLS model reveals a similar pattern, except for the significance on the effects of Maximum BI and total rainy days from June to November. The proportion of the susceptible population may be the most influential risk factor, as indicated by the highest estimated coefficient. We utilize various goodness of fit measures, including R^2^ (pseudo-R^2^ for CPM), deviance residuals, mean square prediction error (MSPE), and mean absolute prediction error (MAPE), to compare the models. The results indicate that the CPM outperforms the OLS model. To confirm this finding, we produce the sample quantile-quantile (QQ) plot of the quantile residuals, a tool often used to assess how well the underlying distribution in regression models fits the original data [42]. The results for OLS and CPM are shown in Fig 4. Points that deviate from the reference line indicate poor model performance and an inappropriate distributional assumption for fitting the considered data. It is clear from the graph that the CPM is appropriate and the OLS model seriously violates the normality assumption. The dispersion and power parameters ($\hat{p}$ and $\hat{\phi }$) for the CPM are estimated at 1.4724 and 1.6947 respectively. These observations suggest that a compound Poisson distribution is suitable for global modeling of dengue transmission intensity. In addition, spatial autocorrelation analysis using Moran’s I statistics shows a significant positive correlation in the residuals of the non-spatial CPM model, indicating the need for spatial modeling in the analysis of the data.

**Table 3:** Parameter estimates for non-spatial CPM and OLS model.

Variable	CPM	OLS
Intercept	-32.4904***	-110.5144***
Logarithm of population density (*X*_1_)	0.3243***	1.2590***
Percent of susceptible population (*X*_2_)	2.5331**	55.9279***
Logarithm of median income (*X*_3_)	-1.1662***	-16.9234***
Maximum BI (*X*_4_)	0.0973***	0.6357
Mean MST days (*X*_5_)	0.2614***	1.7583***
Total rainy days from Jun to Nov (*X*_6_)	0.0927***	0.0419
Longest duration of dryness days (*X*_7_)	0.0736***	0.2779***
Annual maximum temperature (*X*_8_)	0.7295***	4.4708***
Dispersion parameter (*p*)	1.4724***	–
Power parameter (*ϕ*)	1.6947***	–
Pseduo-R^2^/R^2^	0.4678	0.4037
Deviance	1406.3471	80558.4600
MSPE	95.6094	107.1256
MAPE	3.9401	5.4146
Moran’s I	0.1617***	0.2920***

* p-value ≤0.05,

** p-value ≤0.01,

*** p-value ≤0.001

For spatial data analysis, we implement GWCPM to investigate the spatial heterogeneity in data relationships. To assess the effectiveness of GWCPM, we also perform a comparison with GWR. Additionally, we conduct an analysis using GWCPM without boundary constraint on *p* ∉ (1, 2) to allow for analytical flexibility. Both fixed gaussian and adaptive bisquare kernel weighting functions are used for local calibrations. The model results are evaluated by the same criteria considered in Table 3. The metrics in Table 4 show that all GWCPM models (GWCPM_a_, GWCPM_b_, GWCPM_c_, GWCPM_d_) provide better fits than the non-spatial global CPM (see Table 3) and have significantly lower Moran’s I values in the model residuals. This finding is consistent with the point made by Fotheringham et al. [1] regarding the explanatory power of local models and the potential reduction in error autocorrelation by allowing for spatial heterogeneity in the regression parameters. Among the GWCPM analyses, models with adaptive bisquare kernel (GWCPM_a_, GWCPM_b_) fit the dengue data better than those with fixed kernels (GWCPM_c_, GWCPM_d_). The GWCPM with no constraint on the power parameter (GWCPM_b_) is the best model. Such conclusion is further supported by two-way comparisons using the Gini index [43] in Table 5, where the model is chosen based on a “mini-max” argument following Zhang [36]. The GWCPM_b_ has the smallest maximal Gini indices across all rows, indicating better predictive accuracy for dengue transmission intensity.

**Table 4:** Model evaluations between GWCPM and GWR.

Model	GWCPM_a_	GWCPM_b_	GWCPM_c_	GWCPM_d_	GWR_a_	GWR_b_
Bandwidth	161	127	96.100	97.900	0.016	150
Pseudo-R^2^	0.5900	0.6091	0.5429	0.5486	0.5872	0.5817
Deviance	923.2566	829.4115	1006.9908	995.8163	55766.2200	56510.1500
MSPE	73.6556	70.2190	82.1276	82.5384	74.1572	75.1465
MAPE	3.2204	3.0898	3.6307	3.6460	3.1940	3.5684
Moran’s I	0.0827***	0.0503*	0.1554***	0.1530***	0.0889**	0.0840**

GWCPM_a_: GWCPM with adaptive bisquare kernel

GWCPM_b_: GWCPM with *p* ∉ (1, 2) and adaptive bisquare kernel

GWCPM_c_: GWCPM with fixed gaussian kernel

GWCPM_d_: GWCPM with *p* ∉ (1, 2) and fixed gaussian kernel

GWR_a_: GWR with fixed gaussian kernel

GWR_b_: GWR with adaptive bisquare kernel

* p-value ≤0.05,

** p-value ≤0.01,

*** p-value ≤0.001

**Table 5:** Gini indices for two-way comparisons.

	CPM	GWCPM_a_	GWCPM_b_	GWCPM_c_	GWCPM_d_
CPM	−	26.352	28.255	22.191	22.063
GWCPM_a_	-7.867	−	13.062	-7.665	-8.001
GWCPM_b_	-6.322	-5.932	−	-5.405	-5.431
GWCPM_c_	-12.699	19.061	21.435	−	-5.807
GWCPM_d_	-12.566	19.464	21.545	6.465	−

GWCPM_a_: GWCPM with adaptive bisquare kernel

GWCPM_b_: GWCPM with *p* ∉ (1, 2) and adaptive bisquare kernel

GWCPM_c_: GWCPM with fixed gaussian kernel

GWCPM_d_: GWCPM with *p* ∉ (1, 2) and fixed gaussian kernel

**Fig 5:**
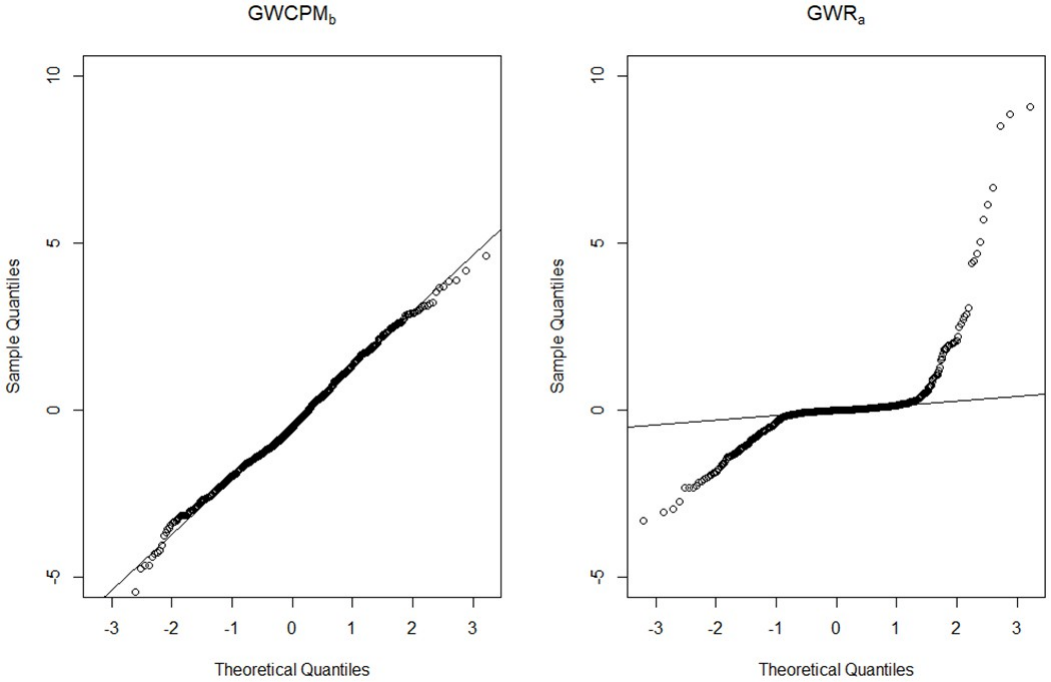
Sample quantile-quantile (QQ) plot of quantile residuals for GWCPM_b_ (left panel) and GWR_a_ (right panel) models.

Compared to the GWR analysis, the GWR model with fixed gaussian (GWR_a_) appears to have slightly worse fitting accuracy than the best GWCPM model (GWCPM_b_) in terms of pseudo-R^2^, MSPE, and MAPE. However, when examining the sample QQ plot of the quantile residuals (Fig 5), the GWR_a_ model fits poorly at both the lower and upper quantiles. This suggests that GWR modeling cannot effectively deal with the substantial proportion of zeros and the skewed heavy-tailed distribution of the positive dengue intensities. It should be emphasized that GWR typically assumes a normal distribution of residuals. This assumption makes it unsuitable for fitting the dengue transmission intensity data with zero-inflation or heavy tails and hence leads to potentially incorrect inferences. In contrast, the sample QQ plot of the quantile residuals for GWCPM_b_ shows that the points mostly lie along the diagonal line, reinforcing the superiority of GWCPM over GWR.

Table 6 gives the five-number summary of the GWCPM_b_ estimation results based on the specifications of unbounded power parameter and an adaptive bisquare kernel weighting scheme. The last column of Table 6 reports the results of the Monte Carlo test for spatial nonstationarity in terms of a total of 999 replications. All the *p*-values in the model are less than the significant level of 5%, which validates the application of the GWCPM model and rules out the possibility of a semiparametric counterpart.

**Table 6:** Parameter estimates of the GWCPM_b_ model.

Variable	Min	Q1	Median	Mean	Q3	Max	p-value
Intercept	-572.0965	-44.8046	-24.1756	-20.8047	0.5341	298.8287	0.001
Logarithm of population density (*X*_1_)	-0.3244	0.0392	0.1359	0.1438	0.2451	0.5835	0.000
Percent of susceptible population (*X*_2_)	-15.0720	-0.8203	5.4671	4.0864	10.0417	15.9329	0.000
Logarithm of median income (*X*_3_)	-7.8557	-2.6566	-1.4768	-1.7633	-0.5264	5.5118	0.000
Maximum BI (*X*_4_)	-0.4789	0.0072	0.0512	0.0747	0.1147	1.3363	0.001
Mean MST days (*X*_5_)	-0.9944	0.0671	0.2252	0.2147	0.4384	1.6400	0.000
Total rainy days from Jun to Nov (*X*_6_)	-2.8399	-0.1303	0.0457	0.0465	0.2407	1.8458	0.000
Longest duration of dryness days (*X*_7_)	-3.4803	-0.1212	0.0421	-0.0259	0.1373	2.2624	0.000
Annual maximum temperature (*X*_8_)	-4.9563	0.0573	0.7767	0.7409	1.2458	11.2805	0.000
Power parameter (*p*)	1.4268	1.5747	1.6328	1.8875	1.8520	2.9999	0.001
Dispersion parameter (*ϕ*)	0.0074	0.1316	0.2659	0.3513	0.5506	1.1335	0.001

**Fig 6:**
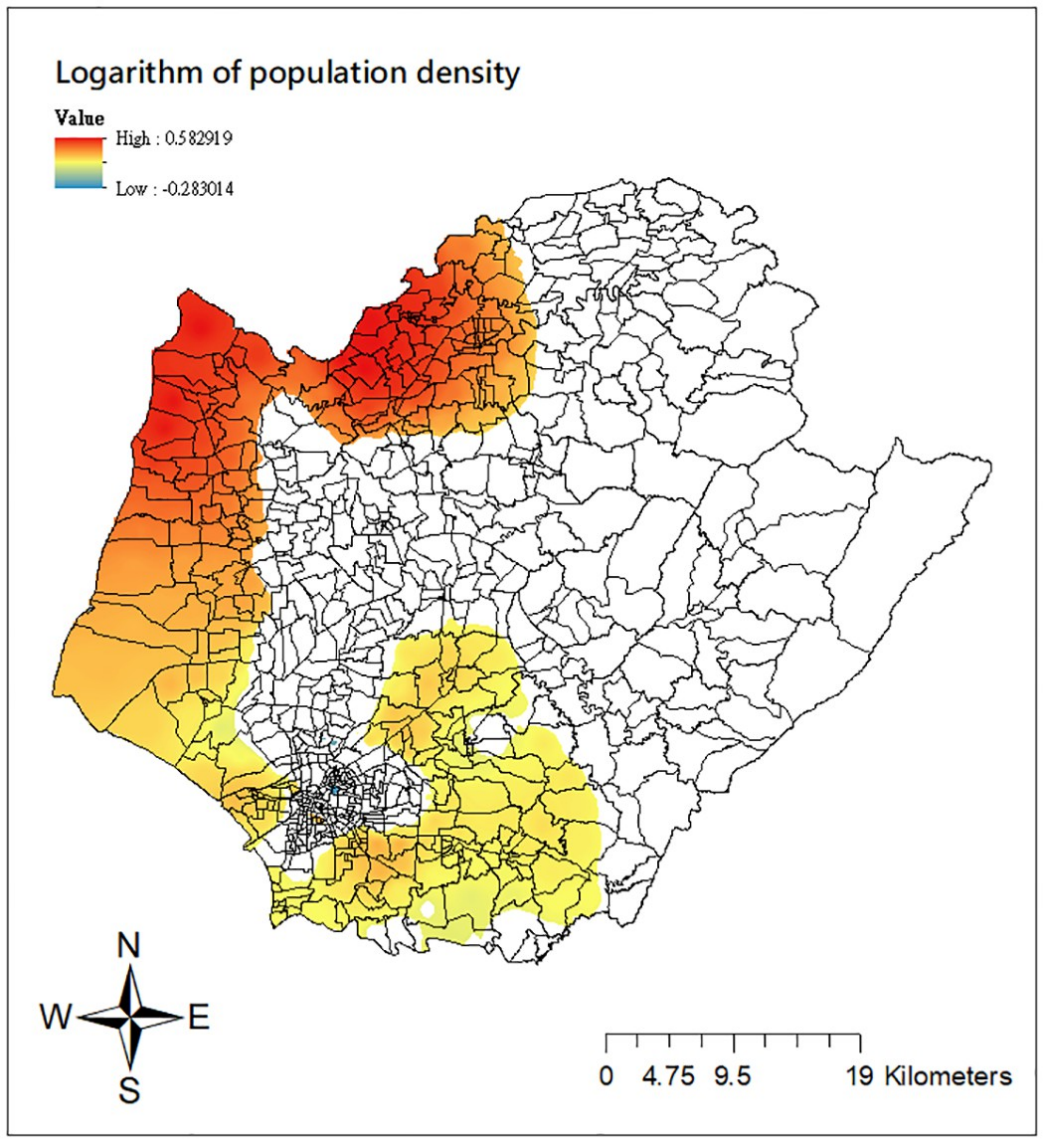
Local estimates of the logarithm of population density from the GWCPM_b_ model. Source: The figure is created by the authors in ArcGIS 10.3. The shapefile used to create the map is publicly available from the Taiwan Ministry of the Interior (https://www.tgos.tw); no copyrighted material was used.

**Fig 7:**
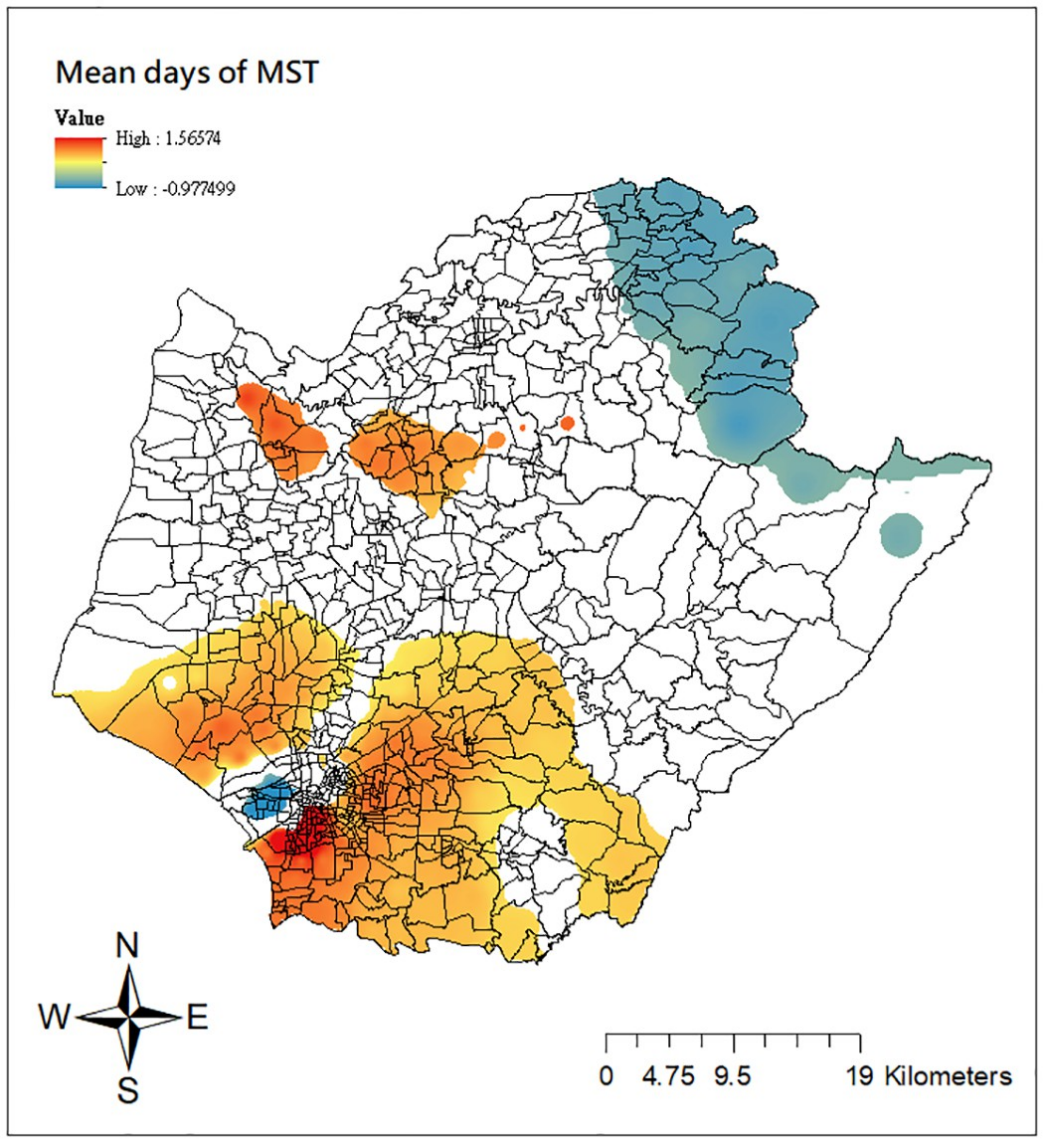
Local estimates of the mean MST days from the GWCPM_b_ model. Source: The figure is created by the authors in ArcGIS 10.3. The shapefile used to create the map is publicly available from the Taiwan Ministry of the Interior (https://www.tgos.tw); no copyrighted material was used.

To gain a deeper understanding of the estimated varying relationships across space, we employ the mapping technique proposed by Matthews and Yang [44] to visualize the results of the GWCPM_b_. Due to space constraints, we opt not to include all maps but only limit two covariates, the logarithm of population density and the mean days of MST. Both of them are known to play important roles on the dengue transmission intensity [39]. Figs 6 and 7 show the spatial distributions of the local parameter estimates for these two variables and only the areas with significant effects at the 5% level are colored. Several findings can be drawn from the figure. First, as shown in Fig 6, larger local estimates of the logarithm of population density are observed in some parts along the coast (e.g., Cigu, Jiangjun, and Beimen districts). This means that ‘Li’ units in the areas with greater population density are more favorable for dengue transmission. Certain ‘Li’ units in northern Tainan (Yanshui, Xinying districts) and on the east side of central districts (e.g., Rende, Guiren, Guanmiao, Xinhua, New Downtown districts) also exhibit a positive but weaker ecological association than coastal regions. Second, in Fig 7, the locations surrounding inner downtown Tainan (e.g., Rende, Guiren, Guanmiao, Yongkang, Xinhua, Xijang, Anan, New Downtown districts) as well as some northwestern areas (Haoying, Xuejia districts), where strong significant positive relationships occur, have a high dengue transmission with increasing mean days of MST. Comparably, the general pattern of negative local estimates in the same graph demonstrates that the intensity of dengue fever transmission is less susceptible to the increased mean days of MST in parts of northern Tainan (Baihe and Dongshan districts). Finally, both maps in Figs 6 and 7 clearly depict the substantial spatial heterogeneity in the relationships between the two covariates and dengue transmission intensity. Significant effects are concentrated on specific parts of Tainan city, rather than being observed uniformly across all ‘Li’ units. Such locality cannot be revealed from the conventional global CPM models.

**Fig 8:**
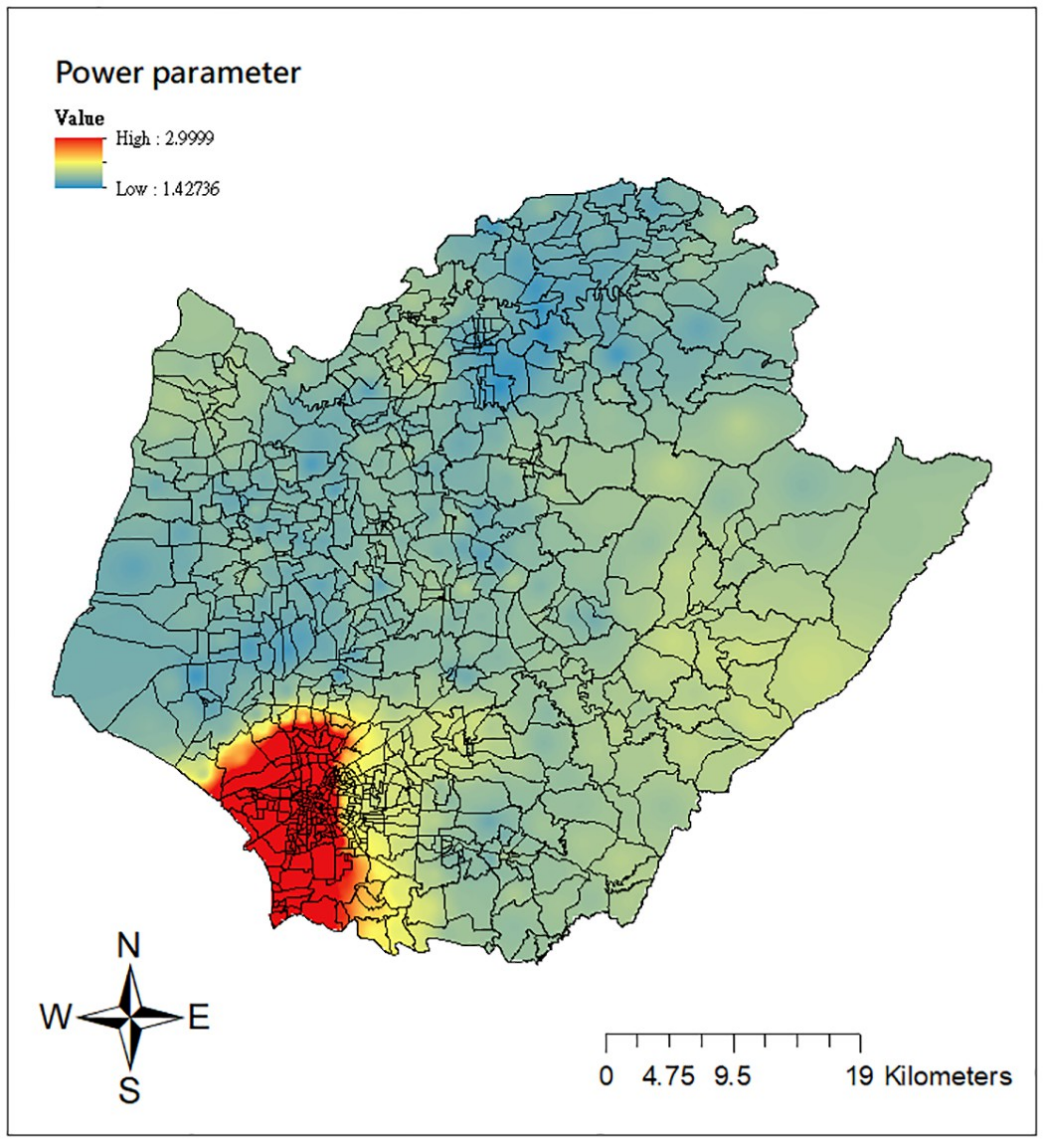
Local estimates of the power parameter from the GWCPM_b_ model. Source: The figure is created by the authors in ArcGIS 10.3. The shapefile used to create the map is publicly available from the Taiwan Ministry of the Interior (https://www.tgos.tw); no copyrighted material was used.

**Fig 9:**
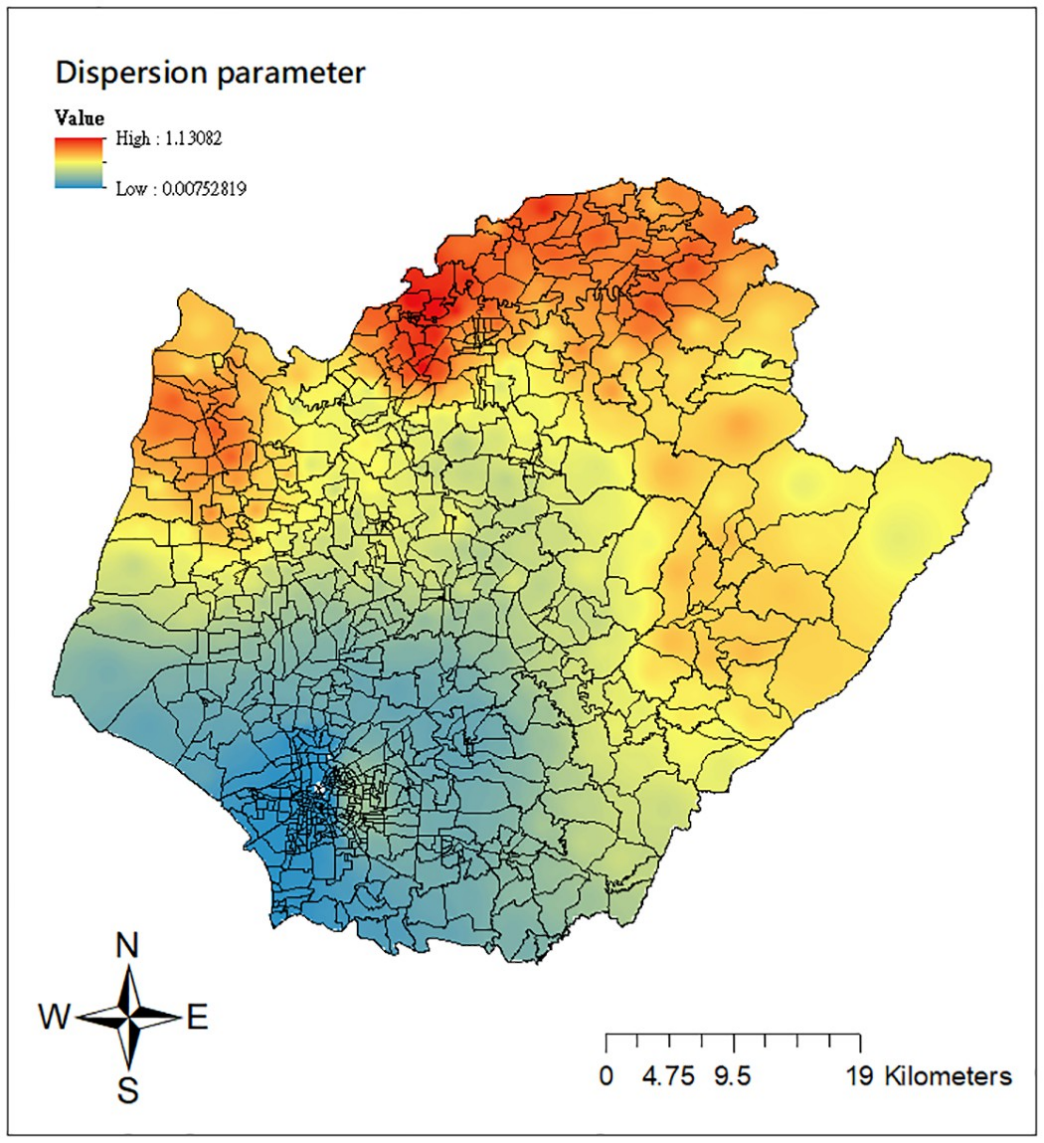
Local estimates of the dispersion parameter from the GWCPM_b_ model. Source: The figure is created by the authors in ArcGIS 10.3. The shapefile used to create the map is publicly available from the Taiwan Ministry of the Interior (https://www.tgos.tw); no copyrighted material was used.

As described in the Methodology section, GWCPM modeling includes the varying power and dispersion parameters to characterize the relationship between the mean and variance at each location, allowing for the capture of various data characteristics of nonnegative continuous outcomes. Thus it is of interest to examine the spatial pattern of such power mean-variance relationships in this case study. The corresponding maps of the estimated power and dispersion parameters computed from the GWCPM_b_ are reported in Figs 8 and 9. We see from Fig 8 that the power index estimates are highest with values close to 3 in the inner downtown area of Tainan city and decreasing outward. The estimates of varying dispersion parameters exhibit a reverse pattern (Fig 9). This phenomenon draws a reciprocal relationship between the power and dispersion parameter, consistent with the CPM literature [26, 36]. We note that the ‘Li’ units, particularly in inner downtown Tainan (West Central, North, East, South, Yongkang, Anping districts) suffered the dengue outbreak in the year 2015, resulting in high dengue transmission intensities with significantly large variations. Therefore, the varying estimates for the power and dispersion parameters correspond to this reality and successfully capture the data characteristics of dengue intensities.

## Conclusion and discussion

GWR has become a widely used approach to explore spatial heterogeneity in data relationships. Despite its popularity, GWR encounters difficulties when modeling nonnegative continuous or so-called semi-continuous outcomes often characterized by significant numbers of zeros and a highly right-skewed distribution of positive values. This paper introduces the GWCPM to resolve such limitations in local spatial modeling approaches. The GWCPM builds upon the traditional non-spatial CPM within the GWR framework. Its primary advantage is the capability to handle the intricate structure and distinctive characteristics of semi-continuous data in local modeling through a single compound Poisson distribution, wherein the distribution variance is expressed as a function of the mean using both dispersion and power index parameters. This power mean-variance relationship, unique to the compound Poisson distribution, offers the flexibility to capture varying levels of data dispersion and tackle issues like zero-inflation and heavy tails. As a result, the GWCPM serves as an effective tool for analyzing spatial data with semi-continuous response variables while allowing for the examination of spatially varying relationships. We also present a semiparametric version of the GWCPM to accommodate spatially invariant parameters, thus enhancing analytical flexibility by controlling for constant factors across different locations. In simulation experiments, we demonstrate the reliable performance of both the GWCPM and its semiparametric counterpart, validating their effectiveness in modeling spatial heterogeneity in semi-continuous data.

Our GWCPM has demonstrated its practical applicability through an empirical analysis of dengue transmission data from Tainan City in southern Taiwan. The results reveal several key findings. On one hand, we find significant spatially varying relationships between dengue transmission and its risk factors, which cannot be captured by global models. For instance, the global CPM indicates a broad positive relationship between mean MST days and dengue transmission, consistent with existing literature [39]. In contrast, GWCPM identifies more localized variations, showing negative associations in parts of northern Tainan and positive associations in southern regions. Such observation echoes that space plays a crucial role in influencing the magnitude and direction of effects among variables of interest (e.g., [29, 39, 45, 46]). On the other hand, the GWCPM analysis underscores the necessity for a place-based approach in developing intervention strategies against dengue transmission at the local level. A one-size-fits-all policy may overlook crucial spatial nuances, as evidenced by the significant nonstationarity retrieved from GWCPM. For example, reducing urbanization in coastal areas of Tainan, where population density is positively associated with transmission intensity, may help control outbreaks. Public health interventions in these regions could benefit from monitoring land cover use and understanding urban dynamics and environmental changes [45–48]. Similarly, climate-related vector control efforts, such as reducing mosquito breeding habitats and deploying mosquito traps, should be prioritized in southern areas with high MST days. This suggestion aligns with previous studies emphasizing the importance of localized modeling in epidemiological or public health research [9, 11, 29]. Moreover, compared to the GWR results, GWCPM effectively handles the dengue transmission data with excessive zeros and severe right skewness. Our proposed method thus offers a robust framework and blueprint for dengue data analysis, enabling more precise assessments of spatially varying relationships.

Some issues warrant further discussion. First, the GWCPM method assumes that the response variable follows a compound Poisson distribution, which belongs to the Tweedie exponential dispersion family and specifies variance proportional to a power of the mean [24]. Should this assumption be violated, the validity of GWCPM may be compromised. While the QQ plot of the quantile residuals offers a straightforward empirical assessment of this assumption, additional sensitivity analyses and simulations are necessary to investigate the effects of dispersion and power index parameters on the model’s performance. In addition, given that GWCPM is developed in the context of GWR, it may be limited in capturing complex nonlinear relationships. This limitation might potentially be addressed by considering the concept of geographically neural network weighted regression (GNNWR) model recently introduced by Du et al. [15], which utilizes a spatially weighted neural network in place of the kernel function. Such a possibility deserves future investigation. It is also worth noting that in spatial data analysis, researchers typically seek to identify locations where the estimated regression coefficients are statistically significant, in addition to parameter estimations and model predictions. Although GNNWR has demonstrated superior fitting accuracy and predictive performance relative to GWR, the main strength of the proposed GWCPM lies in the statistical inference of local model parameters. Specifically, GWCPM considers variance structures and different shapes of the response variable distribution through the power mean-variance specification, ensuring reliable standard error estimation and robust inferences.

Furthermore, current methods for constructing statistical inference in GWCPM rely on asymptotic theory, which sometimes presents problems when the Hessian matrix is not positive definite. In the GWR literature, bootstrapping [49] has been suggested as an inferential framework for GWR-type modeling techniques, including the mixed GWR [33, 50], semiparametric geographically weighted generalized linear models [34], and geographically weighted quantile regression [3]. Therefore, further research in this regard could establish bootstrap inference for GWCPM. Another limitation pertains to the development of the semiparametric GWCPM, as inference on local parameter estimates has not yet been established for the model. Future work could also employ the bootstrap method to address this concern. Additionally, recent studies have adopted bootstrap tests to determine constant coefficients. Similar efforts can be made not only to complement the semiparametric GWCPM framework but also to provide a model selection tool for data analysis. Finally, we should consider an empirical application of the semiparametric GWCPM to illustrate its utility in allowing some regression parameters to remain spatially invariant under appropriate circumstances.

In summary, this study introduces the GWCPM and demonstrates its strength in examining spatially varying coefficients for semi-continuous dependent variables. From both theoretical and empirical standpoints, this approach holds significant potential for advancing geographical analysis and fostering new discussions in the field.
